# 

**Published:** 1887-05

**Authors:** 


### CONTENTS.

COMMUNICATIONS.

Saliva .......... 213
Irregularities .......... 216
Nitrous Oxide .......... 220
Talk on the Materials and Methods used in taking Impressions...... 229
Treatment of Harelip and Cleft Palate .......... 235

PROCEEDINGS.

Michigan State Dental Society. .......... 239
Diet in Acute Febrile Diseases .......... 241
The True Law of Population Based upon Physiology and Psychology 244

SELECTIONS.

Something more about Cocaine .......... 251
Submaxillary Calculus .......... 252
Napelline in Facial Neuralgia .......... 254
Rumination in Man .......... 255

SOCIETY MEETINGS.

Mississippi State Dental Association .......... 255
Missouri State Dental Society .......... 256
Detroit Dental Society .......... 256
Georgia State Dental Society .......... 257
Kentucky State Dental Association .......... 258
State Board of Dental Examiners .......... 259
Southern Dental Association .......... 259
Chicago Dental Society .......... 260
Illinois State Dental Society .......... 260
Northern Ohio Dental Society .......... 261
Wisconsin Dental Society .......... 261
SALMAGUNDI .......... 261

